# An Insight into Wheat Germ Oil Nutrition, Identification of Its Bioactive Constituents and Computer-Aided Multidimensional Data Analysis of Its Potential Anti-Inflammatory Effect via Molecular Connections

**DOI:** 10.3390/life13020526

**Published:** 2023-02-14

**Authors:** Seema Zargar, Tanveer A. Wani, Syed Rizwan Ahamad

**Affiliations:** 1 Department of Biochemistry, College of Science, King Saud University, Riyadh 11495, Saudi Arabia; 2 Department of Pharmaceutical Chemistry, College of Pharmacy, King Saud University, Riyadh 11451, Saudi Arabia

**Keywords:** inflammation, FABP, GC-MS, drug-likeness, *in-silico* analysis

## Abstract

Wheat germ oil (WGO) is the richest source of unexplored antioxidants and anti-inflammatory compounds. In this study, we identified the constituents of WGO by gas chromatography–mass spectrometry (GC-MS). The physicochemical and pharmacokinetic behaviors were evaluated for the top 12 constituents with the common target FABP4. Three fatty acids with significant anti-inflammatory activity were evaluated for their interaction with FABP4 by molecular docking. The molecular mechanisms involved in anti-inflammatory responses were analyzed by various *in-silico* analytical tools and multidimensional data analysis. WGO showed anti-inflammatory activities via FABP4 interacting physically with target genes (77.84%) and by co-expressing with 8.01% genes. Primary targets for inflammatory pathways were PPARα, PPARγ, LPL, LEP, and ADIPOQ, as depicted by gene network enrichment analysis. The key pathways implicated were the metabolism of lipids, PPAR signaling, cellular response to alcohol, oxygen and nitrogen pathway, inflammatory response pathway, and regulation of the inflammatory pathway. The common transcription factors implicated were HNF1, AP2α, CEBP, FOX, STATS, MYC, Zic, etc. In this study, we found that WGO possesses anti-inflammatory potential via FABP4 binding to PPARα, PPARγ, LPL, LEP, and ADIPOQ gene expression by regulatory transcription factors HNF, AP2α, and CEPB.

## 1. Introduction

*Triticum aestivum* (wheat) is an edible products used worldwide and is comprised approximately 80% of endosperm, 15% bran, and 5% germ [1]. The embryo (wheat germ) has been reported to be a good source of antioxidants, carotenoids, polyphenols, and tocopherols [2,3]. Wheat germ is considered highly nutritious and has been incorporated into food or food products such as bread, snacks, and breakfast cereal supplementations. Wheat germ oil (WGO) is produced from the wheat germ by a milling process, and the wheat endosperm contains approximately 10% oil. WGO has broad applications in the food industry as well as in the cosmetic industry [4,5]. WGO is believed to have medical value, and the protein content of WGO has been reported to be a rich source of amino acids such as methionine, threonine, and lysine [6].

Several studies have been conducted to investigate the therapeutic potential of WGO. The effects of WGO have been studied in lipid metabolism, diabetes, and hyperlipidemia management [7,8,9,10]. Fermented wheat germ extract (FWGE) has shown antimetastatic effects in animals including colorectal and ovarian cancer cells [11,12,13,14,15]. Many in vivo trials have been conducted to determine the preventive role of wheat germ on atherosclerosis, hypercholesterolemia, hyperlipidemia, oxidative stress, hepatotoxicity, and insulin resistance [16,17,18,19,20,21]. Many in vitro and in vivo studies have also demonstrated the antioxidant and anti-inflammatory effects of wheat germ oil [21,22,23,24,25]. Inflammation is one of the vital defense mechanisms crucial for homeostasis in acute diseases. Fatty acid trafficking is a dynamic process and affects important cellular functions such as energy source, signaling, metabolomics, and regulation of growth, survival, and inflammatory responses. Lipid chaperones, the fatty acid binding proteins (FABPs), regulate all these processes, potentially serving as a new class of therapeutics for diabetes, obesity, and atherosclerosis [26,27]. To maintain health and prevent diseases, interest has started to develop in the consumption of essential fatty acids (EFA). Consumption of EFA and prevention of heart disease, hypertension, inflammation, cancer prevention, neurological disorders, etc., show a positive correlation with each other [28,29]. Linoleic acid (LA) and α-linolenic acid (ALA) are essential fatty acids found in plant foods and on their consumption, ALA serves as the substrate for the synthesis of eicosapentaenoic acid (EPA) and docosahexaenoic acid (DHA), which are mainly responsible for associated health benefits [30]. The therapeutic benefits of essential fatty acids are dependent on their pharmacokinetics and bioavailability. Before absorption of fatty acids in the gastrointestinal tract, they are subjected to hydrolysis by microflora and then absorbed and distributed. 

A new and promising approach towards new drug discovery and development has been developed with the help of network pharmacology. The network pharmacology when supplemented with system biology and bioinformatics helps to comprehend the effect on various systems including genetics, the protein system, and the information pathways that are involved in biogenesis. All the data obtained are then integrated to build mathematical models to express the effect on the system due to any alterations. An understanding of the various principles by network pharmacology is therefore now considered a new model of drug discovery. Furthermore, in drug discovery, this multidimensional analysis has been used to study various genetic diseases and their complex pathways. Furthermore, computer-aided multidimensional analysis using system biology can be used to elucidate the molecular mechanism of interaction and thus understand the various mechanisms of action [31]. 

This study was designed to explore the active constituents of WGO, since it is rich in essential fatty acids and possesses numerous health benefits. Furthermore, various computer-aided multidimensional data analysis tools and databases were utilized to study the constituents of WGO. The active constituents were studied with respectto their pharmacokinetic behavior and potential anti-inflammatory effects. In addition, the molecular mechanism involved in the anti-inflammatory responses via genetic connections was explored. Molecular docking studies were also conducted to comprehend the interaction between fatty acid binding protein FABP4 and various anti-inflammatory potential links with target genes, transcription factors, and pathways. 

## 2. Material and Methods

### 2.1. Materials

Wheat Germ oil (WGO) was acquired from Wadi Al-Nahil, (Riyadh, Saudi Arabia).

### 2.2. GC-MS Analysis

Agilent GC 7890A combined with a triple-axis detector 5975 C single-quadrupole mass spectrometer was used for GC-MS analysis. The chromatographic column was an Agilent HP 5MS column (30 m × 0.25 mm × 0.25 µm film thickness), with high-purity helium as the gas carrier, at a flow rate of 1 mL/min. The injector temperature was 250 °C and it was equipped with a splitless injector with a ratio of 20:1. The source temperature of MS was set at 230 °C and the Quad temperature was set at 150 °C. The heating program was set at an initial temperature of 40 °C (held for 1 min), then increased to 150 °C at a rate of 10 °C/min held for 1 min, and later increased further to 300 °C at 10 °C min/min for 1 min. The high temperature was maintained for 60 min until the analysis was completed. The scan range was set at 40 to 600 mass ranges at 70 eV electron energy, with a solvent delay of 3 min. A 1 μL sample of WGO was injected directly into the GC-MS system via autosampler. Unknown compounds were identified by comparing the experimental mass spectra with literature data and mass spectral libraries of Wiley and National Institute of Standard and Technology library 2008. The total time required for analyzing a single sample was 29 min. The quadrupole mass analyzer had a scan range of 20–700 amu and a scan speed of 4.0 scans/sec). 

### 2.3. Pharmacokinetics and Physicochemical Characteristics of WGO Compounds

SwissADME software (http://www.swissadme.ch/index.php accessed on 9 February 2023) was used to characterize the components, elucidate their biophysical and pharmacokinetic properties, and ascertain whether the compounds had anti-inflammatory and drug-like activities in humans. The method uses six physicochemical parameters, including lipophilicity, size, polarity, solubility, flexibility, and saturation, to estimate bioavailability radar in order to identify drug-likeness. The ADME properties, including passive human gastrointestinal absorption (HIA), blood–brain barrier (BBB) permeation, and substrate or non-substrate of the permeability glycoprotein (P-gp), as detected positively or negatively in the BOILED-Egg model [32,33]. The rule-of-five pioneer Lipinski (Pfizer) filter has been included into this tool from Lipinski et al. (2001), and it also has an inbuilt feature for the prediction of drug-likeness [34]. Furthermore, the Swiss target prediction tool was used to predict the target genes and proteins affecting, directly or indirectly, three bioactive compounds based on the high quality achieved through GC-MS [32,33]. SwissADME uses canonical smiles of compounds to analyze the target binding.

### 2.4. Gene Interaction Network with Inflammatory Pathways and Genes

The common target gene obtained from SwissADME for all three compounds was analyzed for the interaction network with all associated genes of inflammatory pathways. The Homosapiens option was chosen, and the gene sets interacting with FABP4 (common target) were analyzed for physical interaction, genetic interaction, co-expression, co-localization, shared protein domains, or predicted interactions using GeneMANIA software (https://genemania.org/ accessed on 9 February 2023). The gene set obtained was further refined to inflammatory effects only and the specific genes responsible for inflammatory action were obtained [35,36].

### 2.5. Enrichment Analysis for Anti-Inflammatory Action

Using Metascape, enrichment analysis of the gene list was performed to identify biological pathways and protein–protein interaction enrichments to better understand the role of WGO in treating inflammatory diseases. A total of 122 input IDs (Entrez Gene ID received from Swiss ADME) were automatically converted by Metascape into Human En-trez Gene ID using the EggNOG and Homologene databases. To obtain systems-level datasets, *p*-values were determined through accumulative hypergeometric distributions, and enrichment factors were calculated and employed as filters [37].

### 2.6. Heatmap Cluster Analysis

Using Microsoft^®^ Excel, data were saved in the ".xlsx" file format; each variable (21 physiochemical characteristics) was chosen as a row, and each constituent (12 active constituents) was chosen as a column. Statistical analysis was performed using a MATLAB^®^ coding base and the 3 in 1 "Ana" integrated method as described by Zhao and Wang [38]. The omics dataset from an Excel^®^ file was used to create a 3D heat map and hierarchical cluster heat map.

### 2.7. Molecular Docking Analysis

The active compounds with anti-inflammatory activity hexadecenoic acid, pentadecanoic acid, and linoleic acid were further studied for their interaction with the active site of FABP4 using molecular docking analysis. The structures of the fatty acids were obtained in SDF format from PUB chem with CIDs of 13849, 504166, and 5280450 for pentadecanoic acid, hexadecenoic acid, and linoleic acid, respectively, and then converted to *pdb format using discovery studio. The three-dimensional crystal structures of fatty acid binding protein FABP4 in complex with 6-Chloro-2-isopropyl-4-(3-isopropyl-phenyl)-quinoline-3-carboxylic acid were downloaded in *pdb format from the protein data bank PDB ID:5hz6 (http://www.rcsb.org/pdb) assessed on 15 January 2023. The molecular docking was carried out using Autodock vina [39] and MGL Tools-1.5.6 (Autodock-4) [40]. The docking files were prepared using Autodock-4. The docking parameters were set to default with the grid box set to 30 Å × 30 Å × 30 Å and exhaustiveness was set to 8. All the configurations obtained from docking were physically verified to ensure the correct positioning of the ligand within the binding site. The binding modes were clustered through the root mean square deviation and docking results were compared based on the binding energies of the different conformations. 

## 3. Results

### 3.1. GC-MS Analysis

**Figure 1:**
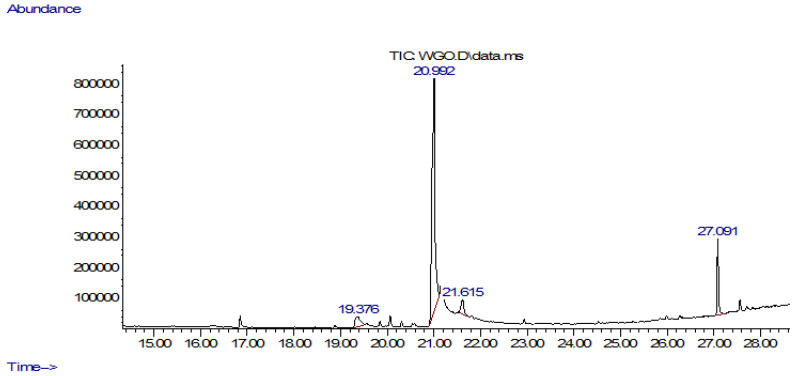
Ion chromatogram obtained from GC-MS.

The top 12 compounds identified by gas chromatography–mass spectrometry (GC-MS) analysis of the wheat germ oil (WGO) are listed in Table 1. The detected compounds accounted for 99% of the total amount and had a peak width of less than 0.463, indicating their good resolution. The total ion chromatogram obtained is shown in Figure 1. The area beneath the peak was proportional to the quality of the compound. Trans-13-Octadecenoic acid (C_18_H_32_O_2_)(99%), squalene (C_12_H_21_N_3_) (93%), and octadecane (C_18_H_38_)(94%) were the most abundant compounds including other fatty acids and terpenoids like Elaidic acid and linoleic acid as depicted by their peak areas, retention time as well as quality (Figure 1). 

### 3.2. SwissADME Analysis

The compounds present in WGO that have been reported to have anti-inflammatory properties are pentadecanoic acid, hexadecenoic acid, linoleic acid, and cyclohexanol [41,42,43]. 

Physicochemical properties are used as a descriptor in many models and provide an opportunity to estimate the ADME properties. As a result, an understanding of whether the evaluated compounds are suitable for further investigation is gained. Drug-likeness is established based on the physicochemical and pharmacokinetic properties of the development compounds. The physicochemical properties of various compounds present in WGO are given in (Table 2). After analyzing the properties of constituents carefully, three bioactive constituents were predicted as good candidates for treating inflammation. All of these were soluble or moderately soluble, hence facilitating drug development activities. According to the results obtained, there were no out-of-range results, indicating that the constituents of WGO have good bioavailability, good gastrointestinal absorption, and are blood–brain barrier permeant. Additionally, the results indicated that WGO is a P-glycoprotein substrate (Table 2). 

### 3.3. Heat Map Analysis

**Figure 2:**
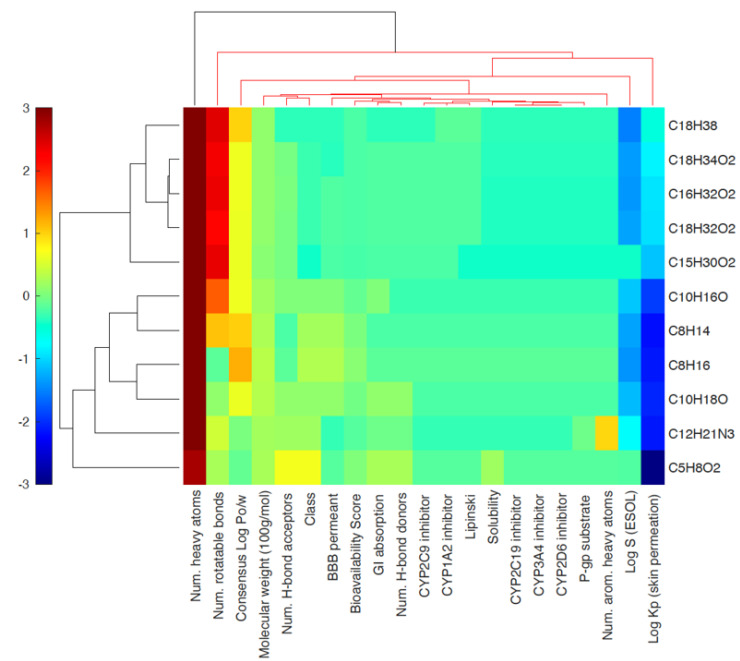
Heat map cluster for each physiochemical variable in a row and each active constituent (11 active constituents) in a column. The red-colored areas showed more associations that are significant, and the lower blue-colored areas represent less association based on the values on the color legend key.

Figure 2 shows the heat map cluster data that were created for the bioactive components of WGO by digitizing SwissADME data on physical and chemical properties to identify significant relationships with the new parameters [38]. Each heat map cell was a numeric count represented by a color code, with darker colors denoting larger linked physiochemical features and drug likelihood. Each of the bioactive components contained a sizable amount of heavy atoms, and some of them possessed sizable rotatable bonds. A majority of the ingredients had good skin-membrane permeability, which may be why WGO protects against skin irritation. In a 3D heat map, each column’s height and color code show the actual positive correlation between each row’s physicochemical parameter and each value.

### 3.4. GeneMANIA Analysis

**Figure 3:**
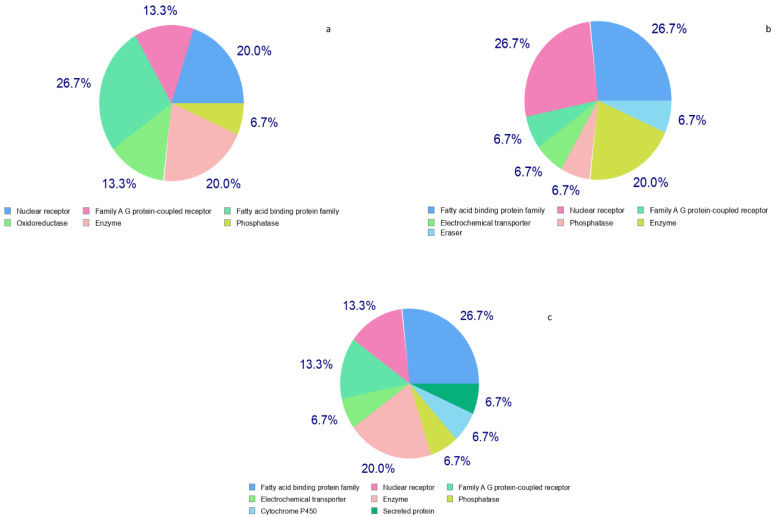
Probability of the bioactive query molecules to the top 15 proteins as targets. (**a**) Hexadecanoic acid/palmitic acid targets; (**b**) linoleic acid targets; (**c**) pentadecanoic acid targets.

**Figure 4:**
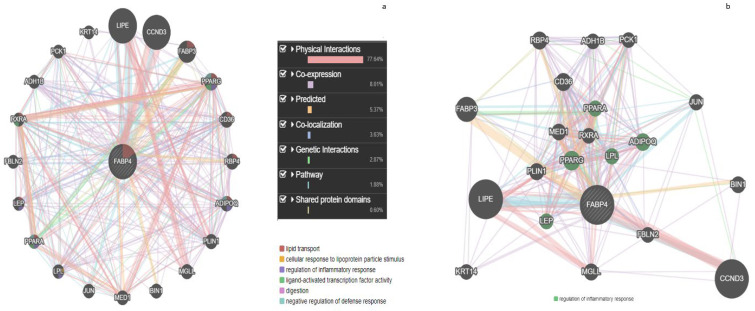
FABP interactions with genes: (**a**) genes involved in inflammatory pathways and other genes; (**b**) genes involved in the inflammatory pathway. The thickness of nodes represents the strength of the interaction. The green highlighted genes are directly involved in inflammation.

The common targets for these three selected constituents were obtained through the target prediction tool available in SwissADME and fatty acid binding protein-4 (FABP4) was chosen as the common target (Figure 3). Data mining through GeneMANIA showed six pathways regulated by FABP4, namely lipid transport, cellular response to lipoprotein particle stimulus, regulation of inflammatory response, ligand-activated transcription factor activity, digestion, and negative regulation of defense response (Figure 4a). The primary gene targets involved in the regulation of inflammatory response by FABP4 were PPARα, PPARγ, LPL, LEP, and ADIPOQ gene regulators as depicted by gene network enrichment analysis. These genes showed 77.6% physical interactions, 8.01% co-expression, and less common co-localization and genetic interactions (Figure 4b).

### 3.5. Metascape Analysis

**Figure 5:**
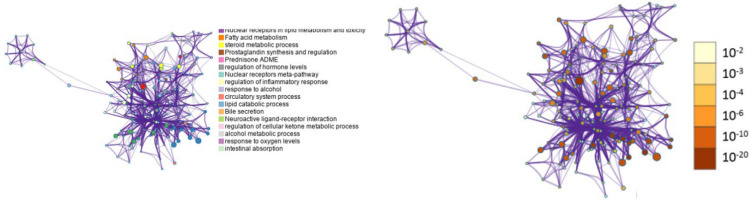
Network of enriched terms: Colored by cluster ID, where nodes that share the same cluster-ID are typically close to each other; colored by *p*-value, where terms containing more genes tend to have a more significant *p*-value. The darker the color, the more statistically significant the node is (legend for *p*-value ranges).

**Figure 6:**
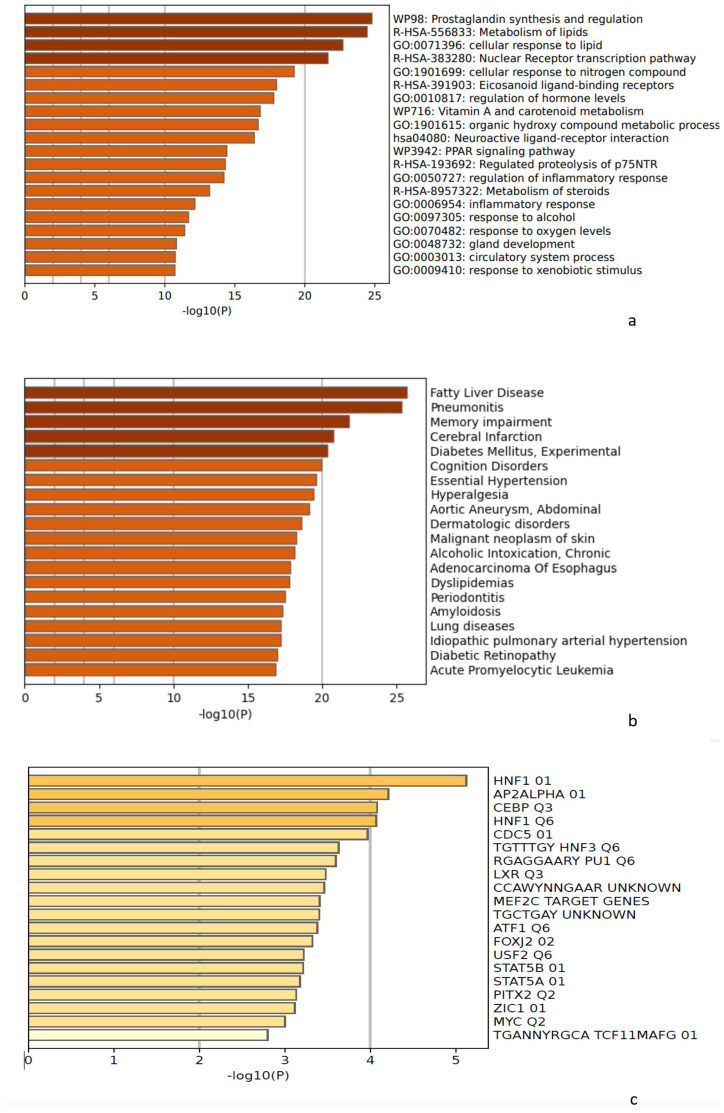
Biological pathways and diseases that may be involved in inflammation are targeted by bioactive constituents. (**a**) Molecular pathways, (**b**) diseases linked to active constituents, and (**c**) transcription factors involved in anti-inflammatory processes.

To analyze the significance of the analyzed genes and transcription factors involved in regulating the expression of anti-inflammatory responses Metascape version 2.5.8 was used to determine enriched biological pathways and protein–protein interaction enrichment analysis. In gene enrichment, a circle node represented each term where its size was proportional to the number of input genes falling under that term, and its color represented cluster identity with nodes of the same color belonging to the same cluster. The darker the color in the legend for the *p*-value range, the more statistically significant the node. The terms that contained more genes had more significant *p*-values (Figure 5). We observed lipid metabolism, nuclear receptor transcription pathway, PPAR signaling pathway, regulation of inflammatory pathway and inflammatory response pathway, response to oxygen and xenobiotics pathways implicated with all three constituents of WGO; therefore, WGO shows protective action through significant FABP4 regulation of all the mentioned pathways (Figure 6a). Through its anti-inflammatory response, WGO can be used in treating fatty liver disease, pneumonitis, memory impairment, cerebral infarction, diabetes, hypertension, dermatologic disorders, malignant neoplasm of skin, lung disease, diabetic retinopathy, acute promyelocytic leukemia (Figure 6b). These diseases were found to be treated by implicating common transcription factors HNF1, AP2α, CEBP, FOX, STATS, MYC, Zic, etc., (Figure 6c) by anti-inflammatory processes. 

### 3.6. Molecular Docking Analysis

Molecular docking was performed on the active sites of the crystal structures of fatty acid binding protein FABP4 (5hz6), with three fatty acids hexadecenoic acid, and linoleic acid having anti-inflammatory activity. The binding energies for the interaction were found to be −5.70, −5.90, and −6.3 Kcal/mole for pentadecanoic acid, hexadecenoic acid, and linoleic acid, respectively.

## 4. Discussion

An important defense mechanism required for maintenance in acute disease situations is inflammation. [44]. However, this process, if not regulated securely by its mediators becomes a reason for targeted destruction and inappropriate repair, leading to persistent host tissue damage with the insurgence of pathologies mainly neurodegenerative diseases, cardiovascular disease (CVD), inflammatory bowel disease (IBD), atherosclerosis, multiple sclerosis, etc. [45]. Different types of pharmacological agents are used to suppress inflammation-associated disorders [46], but these agents are associated with undesirable complications and adverse effects. Therefore, there is a need to explore natural alternatives with anti-inflammatory potential and satisfactory tolerability as a long-term effective strategy to fight against inflammation [47,48]. Wheat germ oil (WGO) is a unique food supplement with concentrated nutrient efficiency and remarkable antioxidant and anti-inflammatory functions [24]. Avemar is a non-toxic wheat germ extract and was approved for clinical use in Hungary in 1998 and later in the Czech Republic, Bulgaria, and Romania, as a medical nutrient for cancer patients [12]. Our previous study investigated the impact of WGO on TAA-induced mice inflammatory and enzymatic markers IL-1β and IL-6 in the liver and kidney tissues of the mice. WGO-treated mice showed restoration of elevated levels significantly in both the liver and kidney relative to the control group [22,23,24,49]. Consistent with our study, previous studies have also shown a protective effect of WGO against streptozotocin, deltamethrin, and chlorpyriphos. WGO has several pharmaceutical applications, since it is considered a protectant against hepatotoxicity and inhibits lipid peroxidation that is induced by various superoxide-free radical producers [18,50]. In this study, we identified the bioactive constituents and used in silico approaches to assess the drug-likeness of WGO to identify if it can serve as a natural therapeutic agent in unregulated inflammatory responses. Information gathered about the ADME of a drug during the discovery phase decreases failure due to pharmacokinetics issues during the early developmental phases. The ADME is predicted using computer-aided technologies as an alternative to the experimental procedures. These alternate methodologies have drastically reduced the cost of drug-designing research. The physicochemical properties are essential to identifying the suitability or drug-likeness of new drug candidates for oral administration. A variety of software has been developed to predict the pharmacokinetics of drugs based on their molecular structure. The SwissADME is one such tool that is used to predict drug-likeness based on the physiochemical and pharmacokinetics properties of new drug candidates. It provides information about bioavailability, solubility, metabolism, and distribution [51]. Three bioactive constituents—pentadecanoic acid, hexadecenoic acid, and linoleic acid—were predicted to be good candidates for treating inflammation with respect to all of the above-mentioned criteria for drug-likeness. All these constituents were soluble or moderately soluble, which greatly facilitates drug development activities and the ease of preparing formulation [52]. All these parameters are the qualitative analysis used to assess the chance of a molecule becoming an oral drug [33]. In addition, the results suggest that all of the bioactive constituents are P-glycoprotein substrates and can cross the blood–brain barrier. P-glycoprotein (P-gp) efflux assay is an integral part of discovery screening for drugs requiring blood–brain barrier permeation [53]. The common target binding to bioactive constituents of WGO was FABP4, which plays an important role in inflammation. Previous studies have reported that suppressing FABP4 decreases inflammation [54]. In this study, we found that all our constituents could bind with FABP4, hence decreasing inflammation. The protective anti-inflammatory effect of WGO was reported in our previous study, but the molecular mechanism of this anti-inflammatory action concerning genes, transcription factors, pathways, and its physicochemical and pharmacokinetic properties were not elucidated [24]. In this study, we closely observed the molecular mechanisms involved in WGO’s anti-inflammatory activity. The chemical analysis of the WGO was undertaken using GC/MS, and then potential targets having anti-inflammatory responses and drug-likeness were identified. 

The gene function, analysis of gene lists, and gene prioritization for functional assays can be performed with the help of GeneMANIA. It provides a user-friendly interface to test hypotheses regarding the above parameters. It functions by finding similar genes as the query submitted to it with its available resources of genomics and proteomics. It is believed to have a very high accuracy in the prediction algorithm and hence provides a useful tool for scientists [55]. It was observed that 20 genes related to the inflammation pathway were regulated by WGO (Figure 4a), and all of them bound to FABP4. More specifically, PPARα, PPARγ, LPL, LEP, and ADIPOQ were identified as key target inflammatory genes regulated by WGO. In concurrence with this study, many previous studies linked FABP4 overexpression to inflammation and its inhibition or knockdown to protect against inflammation. Recently, a study reported that the FABP4 knockdown suppresses inflammation, apoptosis, and extracellular matrix degradation in IL-1β-induced chondrocytes by activating PPARγ to regulate the NF-κB signaling pathway. PPAR*α* and *PPAR-γ* have been reported to regulate inflammatory processes by inhibiting inflammatory gene expression [54,56,57]. PPAR*γ* is known to decrease inflammation in activated macrophages by interfering with NF-*κ*B signaling pathways [58]. Furthermore, evidence suggests the relation of focal arterial expression of LpL to aortic macrophage levels and inflammatory processes giving evidence for new therapeutic approaches on lowering abnormal plasma triglyceride-rich lipoproteins, reducing arterial lipid deposition, and suppressing adverse inflammatory events [59]. Similarly, adiponectin (anti-inflammatory), leptin (pro-inflammatory) adipokines and their ratio (ADIPOQ/Lep) reflect inflammatory burden [60]. Transcription factors perform critical functions by modulating gene expression; therefore, we assessed the interactions of the above genes with transcription factors in relation to inflammatory pathways targeted by WGO. HNF, AP2α, and CEPB were the most important transcription factors regulating these genes. HNF transcription regulates albumin gene expression. Decreases in plasma albumin during acute inflammation are mediated by pro-inflammatory cytokines; IL-6 [12], and TNF-α are in turn regulated by CEPB [61]. Activator Protein 2 alpha (AP2α) tumor suppressor *suppresses* C/CAAT enhancer binding protein alpha (C/EBPα*)*, a transcription factor involved in cell cycle control and cellular differentiation [62]. 

Molecular docking was performed for the various components presents in the WGO, which included pentadecanoic acid, hexadecenoic acid, and linoleic acid FABP4 [63,64,65]. Among the studied compounds, linoleic acid was found to have the highest affinity, followed by pentadecanoic acid and hexadecenoic acid.

**Figure 7:**
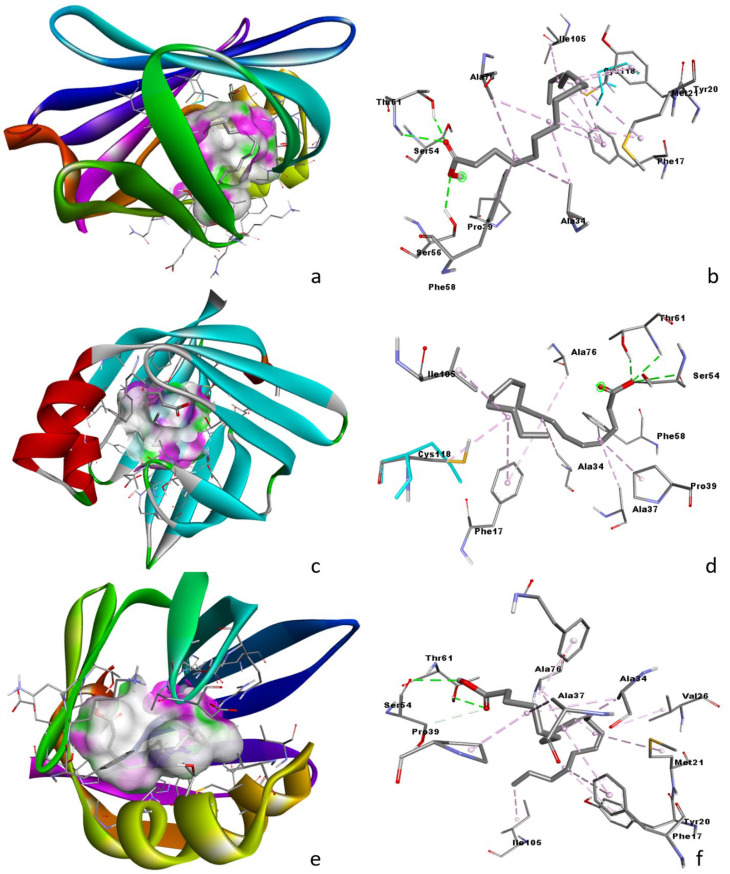
Three-dimensional docking conformation of FABP4 with pentadecanoic acid (**a**,**b**); hexadecenoic acid (**c**,**d**); linoleic acid (**e**,**f**).

**Figure 8:**
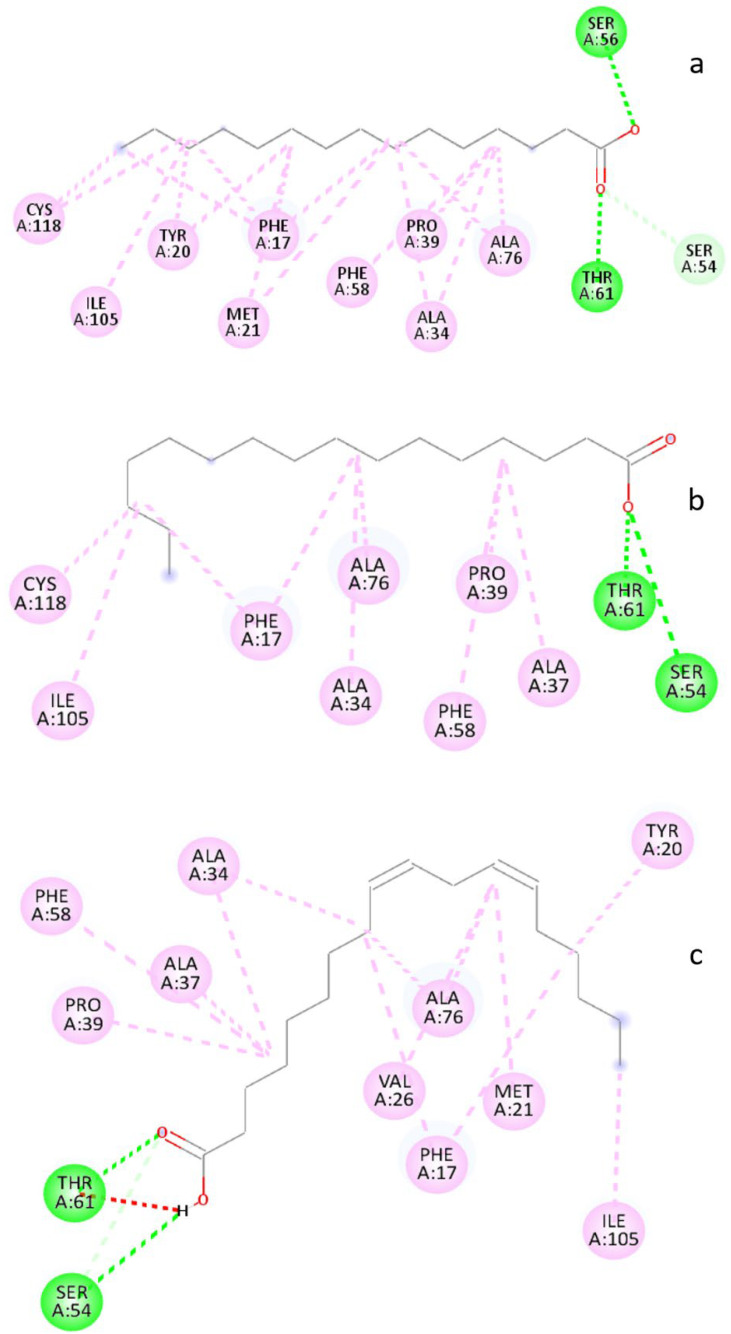
Two-dimensional conformation of docking interaction of FABP4 with pentadecanoic acid (**a**); hexadecenoic acid (**b**); linoleic acid (**c**).

The analysis of the binding sites shows the FABP4 binds to pentadecanoic acid through hydrogen bonds, and hydrophobic interactions (Figure 7a,b and Figure 8a). Three hydrogen bonding interactions were observed between pentadecanoic acid and FABP4; Ser56: pentadecanoic acid: O1 (2.71 Å), Thr61: pentadecanoic acid: O2 (2.91 Å) and Thr61: pentadecanoic acid: O2 (2.07 Å). Various other alkyl and π-alkyl interactions were also observed with Ala34, Pro39, Ala76, Cys118, Met21, Phe17, Tyr20, and Phe58. The binding affinity for the interaction was found to be −5.90 Kcal/mole.

The docking of hexadecenoic acid with FABP4 revealed hydrogen bonds and hydrophobic interactions (Figure 7c,d and Figure 8b). The interactions involved four hydrogen bonds between hexadecenoic acid and FABP4; Ser54: hexadecenoic acid: O1 (3.17 Å), Ser54: hexadecenoic acid: O1 (3.51 Å), Thr61: hexadecenoic acid: O1 (2.68 Å), Thr61: hexadecenoic acid: O1 (2.01 Å). In addition to these hydrogen bonds, six alkyl bonds (Ala34, Ala37, Pro39, Ala76, Cys118, and Ile105) and three pi-alkyl bonds (Phe17, and Phe58) were also observed. The binding affinity for the interaction was found to be −5.70 Kcal/mole.

Furthermore, the docking interaction between linoleic acid and FABP4 revealed three hydrogen bonds and 13 hydrophobic bonds, which included nine alkyl bonds and four pi-alkyl bonds (Figure 7e,f and Figure 8c). The hydrogen bonds were observed between Ser54: linoleic acid: O2 (3.62 Å), Ser54:O: linoleic acid: H52 (2.80 Å) and Thr61: linoleic acid: O2(2.38 Å). The hydrophobic bonds alkyl was formed with (Ala34, Ala37, Pro39, Ala76, Val26, Met21, and Ile105) and 4 pi-alkyl bonds were formed with (Phe17, Tyr20and Phe58) were also observed. The binding affinity for the interaction was found to be −6.30 Kcal/mole. This study gives a more in depth molecular mechanism of the genetic connections necessary to understand the precise role of WGO’s protective anti-inflammatory action. 

## 5. Conclusions

In this work, the chemical makeup of bioactive molecules was analyzed using GC/MS in order to determine the anti-inflammatory potential of WGO. A computer-aided multidimensional analysis was used to identify and assess WGO components that have anti-inflammatory effects. This analysis considered physical interactions, genetic interactions, co-expression, co-localization, shared protein domains, and predicted interactions of the complex pathways at play. It was postulated that the regulatory transcription factors HNF, AP2, and CEPB activate the genes PPAR, PPAR, LPL, LEP, and ADIPOQ via FABP4 binding to those genes. Additionally, molecular docking was used to examine the interactions between the three possible constituents pentadecanoic acid, hexadecenoic acid, and linoleic acid and FABP4. The findings indicate the fact that all three of the bioactive ingredients that were studied bind to FABP4, which is the target protein. In subsequent studies, a greater emphasis will be placed on elucidating the molecular underpinnings of WGO’s anti-inflammatory activities. 

## Figures and Tables

**Table 1:** Major constituents of WGO and their chemical composition by GC-MS analysis.

S.No	RT (min)	Area (Ab*s)	Peak Width 50% (min)	Compound	Quality	Mol Weight (amu)
1	12.136	765,017	0.463	2,4-Decadienal, (E,E)-	80	152.12
2	16.863	95,170	0.189	Cyclopentane, 1,1,3-trimethyl-	53	112.125
3	18.906	43,909	0.189	1,4-Heptadiene, 3,3,6-trimethyl-	12	138.141
4	19.397	337,266	0.265	Pentadecanoic acid	45	242.225
5	19.586	48,474	0.217	n-Hexadecanoic acid	60	256.24
6	19.842	43,328	0.123	2-Butyn-1-ol, 4-methoxy-	10	100.052
7	20.314	42,111	0.132	Cyclohexanol, 5-methyl-2-(1-methylethenyl)-[1R-(1.alpha.,2.beta.,5.alpha.)]-Cyclohexanol	47	154.136
8	21.005	4,075,785	0.255	trans-13-Octadecenoic acid	99	282.256
9	21.288	724,675	0.369	9,12-Octadecadienoic acid (Z,Z)-	99	280.24
10	22.933	42,472	0.113	Piperazine, 1-[2-(2,5-dimethyl-1H-pyrrol-1-yl)ethyl]-	30	207.174
11	27.103	593,470	0.265	Squalene	93	410.391
12	27.567	84,611	0.123	Octadecane	94	254.297

**Table 2:** Physicochemical properties for drug-likeness and physicochemical inspections of compounds isolated from WGO obtained from SwissADME.

NAME	C_10_H_16_O	C_8_H_16_	C_8_H_14_	C_15_H_30_O_2_	C_16_H_32_O_2_	C_5_H_8_O_2_	C_10_H_18_O	C_18_H_34_O_2_	C_18_H_32_O_2_	C_12_H_21_N_3_	C_18_H_38_
Molecular weight (100 g/mol)	1.5223	1.1221	1.102	2.424	2.5742	1.0012	1.5425	2.8246	2.8045	2.0732	2.5449
Num. heavy atoms	11	8	8	17	18	7	11	20	20	15	18
Num. arom. heavy atoms	0	0	0	0	0	0	0	0	0	5	0
Num. rotatable bonds	6	0	3	13	14	1	1	15	14	3	15
Num. H-bond acceptors	1	0	0	2	2	2	1	2	2	2	0
Num. H-bond donors	0	0	0	1	1	1	1	1	1	1	0
Consensus Log Po/w	2.85	3.07	2.87	4.84	5.2	0.18	2.42	5.64	5.45	1.28	7.18
Log S (ESOL)	−2.44	−2.81	−2.49	0	−5.03	−0.05	−2.59	−5.41	−5.05	−1.67	−6.33
Solubility	3.67 × 10^−3^	1.55 × 10^−3^	3.26 × 10^−3^	0	9.35 × 10^−6^	8.95 × 10^−1^	2.58 × 10^−3^	3.85 × 10^−6^	8.87 × 10^−6^	2.16 × 10^−2^	4.67 × 10^−7^
Class	1	1	1	0	0.5	2	1	0.5	0.5	2	0
GI absorption	1	0	0	1	1	1	1	1	1	1	0
BBB permeant	1	1	1	1	1	0	1	0	1	0	0
P-gp substrate	0	0	0	0	0	0	0	0	0	1	0
CYP1A2 inhibitor	0	0	0	1	1	0	0	1	1	0	1
CYP2C19 inhibitor	0	0	0	0	0	0	0	0	0	0	0
CYP2C9 inhibitor	0	0	0	1	1	0	0	1	1	0	0
CYP2D6 inhibitor	0	0	0	0	0	0	0	0	0	0	0
CYP3A4 inhibitor	0	0	0	0	0	0	0	0	0	0	0
Log Kp (skin permeation)	−4.92	−4.42	−4.54	−3.07	−2.78	−7.3	−5.15	−2.6	−3.05	−7.01	−1.2

## Data Availability

Data will be available on request to the corresponding author.
